# Higher anti-depressant dose and major adverse outcomes in moderate chronic kidney disease: a retrospective population-based study

**DOI:** 10.1186/1471-2369-15-79

**Published:** 2014-05-10

**Authors:** Varun Dev, Stephanie N Dixon, Jamie L Fleet, Sonja Gandhi, Tara Gomes, Ziv Harel, Arsh K Jain, Salimah Z Shariff, Davy Tawadrous, Matthew A Weir, Amit X Garg

**Affiliations:** 1 Schulich School of Medicine, Western University, London, Canada; 2 Division of Nephrology, Western University, London, Ontario, Canada; 3 Department of Epidemiology and Biostatistics, Western University, London, Ontario, Canada; 4 Institute for Clinical Evaluative Sciences, Ontario, Canada; 5 Leslie Dan Faculty of Pharmacy, University of Toronto, Toronto, Ontario, Canada; 6 Keenan Research Centre, Li Ka Shing Knowledge Institute, Toronto, Ontario, Canada; 7 Division of Nephrology, University of Toronto, Toronto, Ontario, Canada; 8 London Kidney Clinical Research Unit, Room ELL-101, London Health Sciences Centre, 800 Commissioners Road East, London, Ontario N6A 4G5, Canada

**Keywords:** Anti-depressant, Delirium, Aged, Chronic renal insufficiency, Cohort studies, Risk

## Abstract

**Background:**

Many older patients have chronic kidney disease (CKD), and a lower dose of anti-depressants paroxetine, mirtazapine and venlafaxine is recommended in patients with CKD to prevent drug accumulation from reduced elimination. Using information available in large population-based healthcare administrative databases, we conducted this study to determine if ignoring the recommendation and prescribing a higher versus lower dose of anti-depressants associates with a higher risk of adverse events.

**Methods:**

We conducted a population-based cohort study to describe the 30-day risk of delirium in older adults who initiated a higher *vs.* lower dose of these three anti-depressants in routine care. We defined delirium using the best proxy available in our data sources - hospitalization with an urgent head computed tomography (CT) scan. We determined if CKD status modified the association between anti-depressant dose and outcome, and examined the secondary outcome of 30 day all-cause mortality. We used multivariable logistic regression analyses to estimate adjusted odds ratios (relative risk (RR)) and 95% confidence intervals.

**Results:**

We identified adults (mean age 75) in Ontario who started a new study anti-depressant at a higher dose (n = 36,651; 31%) or lower dose (n = 81,160; 69%). Initiating a higher *vs.* lower dose was not associated with an increased risk of hospitalization with head CT (1.09% *vs.* 1.27% (adjusted RR 0.90; 95% CI, 0.80 to 1.02), but was associated with a lower risk of all-cause mortality (0.76% *vs.* 0.97% RR 0.82; 95% CI, 0.71 to 0.95). Neither of these relative risks were modified by the presence of CKD (*p* = 0.16, 0.68, respectively).

**Conclusions:**

We did not observe an increase in two adverse outcomes when study anti-depressants were initiated at a higher dose in elderly patients with moderate CKD. Contrary to our hypothesis, the 30-day risk of mortality was lower when a higher versus lower dose of anti-depressant was initiated in these patients, a finding which requires corroboration and further study.

## Background

Anti-depressants are among the most widely prescribed medications worldwide and depression is the most common mental health problem in the elderly [1-5]. The prevalence of depression in elderly individuals with chronic kidney disease (CKD) is estimated at 15 – 30% [6]. Older adults have age-related changes in drug metabolism that can increase their risk of adverse drug effects, a finding particularly true for many anti-depressants [7-9]. Up to 30% of these older adults also have CKD, which can cause drug accumulation through reduced elimination, further increasing the risk of adverse events [10,11]. A number of pharmacokinetic studies have demonstrated that the serum half lives of three common anti-depressants (paroxetine, mirtazapine or venlafaxine) are significantly prolonged in individuals with CKD [6,12-14]. Additionally, several case reports link the use of these three anti-depressants to delirium, a state of acute confusion that can lead to hospitalization and death [15-18]. Thus, in CKD, use of lower doses of these three anti-depressants is recommended in drug prescribing references (recommendations summarized in Table 1). Using information available in large population-based healthcare administrative databases, we conducted this study in older adults to determine if ignoring the recommendation and initiating a higher *vs.* lower dose of three study anti-depressants (paroxetine, mirtazapine or venlafaxine) associates with a higher risk of adverse events. The two outcomes assessed in our data sources were 30-day risk of delirium (assessed through a proxy of hospitalization with an urgent head computed tomography (CT) scan) and mortality. We also examined if the presence of CKD modified the association between anti-depressant dose (higher v. lower) and outcome.

**Table 1:** Anti-depressant dosing in popular drug prescribing references

	Higher dose (mg/day)*	Lower dose (mg/day)*	**UpToDate recommendation**[19]**-**[21]**]**	**Compendium of pharmaceuticals and specialties**[22]**-**[24]**]**	**Systematic review**[6]**]**
**Paroxetine**	> 20	≤ 20	• 20 – 50 mg/day	• 20 – 50 mg/day	• 20 – 50 mg/day
			• Reduce by 50% with CrCl < 30 ml/min	• Initiate at 10 mg/day with CrCl < 40 ml/min	• Initiate at 10 mg/day with eGFR < 60 ml/min
**Mirtazapine**	> 20	≤ 20	• 15 – 45 mg/day	• 15 – 45 mg/day	• 15 – 45 mg/day
			• Use with caution with CrCl < 40 ml/min	• Use with caution	• Use with caution
					• Initiate at 15 mg/day with eGFR < 30 ml/min
**Venlafaxine**	> 37.5	≤ 37.5	• 75 – 225 mg/day	• 75 – 225 mg/day	• 75 – 225 mg/day
			• Reduce by 25 – 50% with CrCl 10 – 70 ml/min	• Reduce by 25 – 50% with GFR 10–70 ml/min	• Reduce to 37.5 mg/day with eGFR < 30 ml/min
				• Initiate at 37.5 mg/day	

*As defined in this study.

*Abbreviations: CrCl* Creatinine clearance, *eGFR* estimated glomerular filtration rate.

## Methods

### Setting and study design

Residents of the province of Ontario, Canada have universal access to hospital care and physician services. Those 65 years of age or older, representing approximately 2 million individuals in 2012, also have universal prescription coverage [25]. All health care encounters in Ontario are recorded in linked, de-identified databases at the Institute for Clinical Evaluative Sciences (ICES). We conducted a retrospective, population-based cohort study using six of these healthcare databases. We conducted this study according to a pre-specified protocol that was approved by the research ethics board at Sunnybrook Health Sciences Centre (Toronto, Canada). The reporting of this study follows guidelines for observational studies (detailed in Additional file 1: Figure S1) [26].

### Data sources

We ascertained baseline characteristics, drug use and dose, and outcome data using six linked healthcare databases. Demographic and vital status information on all Ontario residents who have ever been issued a health card is recorded in the Ontario Registered Persons Database (RPDB). Detailed diagnostic and procedural information on all hospital admissions and emergency room visits is recorded in the Canadian Institute for Health Information Discharge Abstract Database (CIHI-DAD) and the National Ambulatory Care Reporting System (NACRS), respectively. Health claims for inpatient and outpatient physician services are recorded in the Ontario Health Insurance Plan database (OHIP). Outpatient prescription drug information including the dispensing date, quantity of pills, dose, and number of days supplied is accurately recorded in the Ontario Drug Benefit Program database (ODB), with an error rate less than 1% [27]. Lastly, the ICES Physician Database (IPDB) contains information on all physicians in Ontario such as sub-specialty, education, location and demographics.

Among a subpopulation of patients with prescriptions filled in Southwestern Ontario, we also obtained baseline serum creatinine values from two linked laboratory datasets: Gamma-Dynacare, a large outpatient provincial laboratory provider and Cerner® (Kansas City, Missouri, USA), an electronic medical record database containing inpatient, outpatient, and emergency department laboratory values for 12 hospitals in Southwestern Ontario [28]. The most recent serum creatinine was obtained in the year prior to the study anti-depressant prescription (median 94 days prior to the prescription). These data sources have been used previously to study drug safety [29-31]. With the exception of anti-depressant prescriber specialty and income quintile (missing in 13.5% and 0.3% of patients, respectively), the databases were complete for all variables used in this study.

### Patients

We established a cohort of all older adults in Ontario who had evidence of a new outpatient prescription for a study anti-depressant (defined as no prescriptions for any type of study or non-study anti-depressant in the prior six months) between April 1^st^, 2002 and December 31^st^ 2011 (n = 169,435). The three study anti-depressants were paroxetine, mirtazapine, and venlafaxine. Patients with multiple eligible prescriptions could only enter the cohort once, and the date of anti-depressant initiation served as the patient’s index date (cohort entry date; start of follow-up). We assessed baseline demographic characteristics, co-morbid conditions (5 years prior to index date) and concurrent drug therapy (180 days prior to index date) among all individuals. We excluded the following anti-depressant users from the analysis: those in the first year of eligibility for prescription drug coverage (age 65 years) to avoid incomplete medication records (n = 12,588); those who were discharged from hospital in the two days before their index date to ensure that prescriptions were new outpatient anti-depressant prescriptions (as in Ontario, patients continuing an antidepressant treatment initiated in hospital would have their oral outpatient antidepressant prescription dispensed on the same day or the day after hospital discharge) (n = 3,833); those living in long-term care facilities because some residents chronically experience bouts of confusion (n = 30,360); those with end-stage renal disease since treatments such as dialysis alter anti-depressant pharmacokinetics unpredictably [14] (n = 1,522); and those who received more than one type of anti-depressant on their index date to allow comparison of mutually exclusive exposure groups (n = 3,321). A total of 117,811 patients were included in the final analysis.

We identified individuals with moderate CKD using an algorithm of diagnosis codes validated in our region for older adults [32]. The algorithm identifies a group of patients with a low GFR by the Chronic Kidney Disease Epidemiology Collaboration (CKD-EPI) formula. It identified patients with a median estimated glomerular filtration rate (eGFR) of 38 mL/min per 1.73 m^2^ (interquartile range 27 to 52), whereas its absence identified patients with a median eGFR of 69 mL/min per 1.73 m^2^ (interquartile range 56 to 82).

### Anti-depressant dose

To align with recommendations in drug prescribing references, a higher dose of anti-depressant was defined as > 20 mg/day of paroxetine, > 20 mg/day of mirtazapine, or > 37.5 mg/day of venlafaxine (Table 1). A lower dose of anti-depressant was defined as ≤ 20 mg/ day of paroxetine, ≤ 20 mg/day of mirtazapine, or ≤ 37.5 mg/day of venlafaxine (Table 1).

### Outcomes

As most reported anti-depressant related delirium occurs within the first few weeks of drug initiation, we followed all individuals for 30 days after first anti-depressant use for two pre-specified outcomes [33]. Our primary outcome was hospitalization with evidence of an urgent head CT scan. We used this as a proxy for the presence of acute central nervous system disturbance (i.e. delirium), as many patients in Ontario undergo such diagnostic imaging when presenting to hospital with acute confusion. Unlike diagnostic codes for acute delirium, the receipt of a head CT scan is well coded in our data sources because they are associated with physician reimbursement [34]. To focus on urgent imaging conducted for acute reasons at the time of hospital admission, we only considered head CT scans performed in the first five days of a hospital admission, or in the emergency department assessment preceding the hospital admission. We expected urgent head CT scans conducted for reasons unrelated to anti-depressant dosing (e.g. stroke, headache) to occur at a similar frequency in higher and lower dose groups, not impacting estimates of difference in risk between the two groups. Our secondary outcome was all-cause mortality which is well coded in our data sources [35].

### Statistical analysis

We compared baseline characteristics between those prescribed higher *vs.* lower daily doses of study anti-depressants using standardized differences. This metric describes differences between group means relative to the pooled standard deviation and indicates a meaningful difference if greater than 10% [36]. We used multivariable logistic regression analyses (PROC LOGISTIC; SAS Institute, Cary, North Carolina) to estimate adjusted odds ratios and 95% confidence intervals. We adjusted for 15 potential confounders: antidepressant type, age, sex, year of cohort entry, modified Charlson score (a co-morbidity index), and concurrent medication use (anticonvulsants, gabapentin, antipsychotics, barbiturates, benzodiazepines, histamine2-receptor antagonists, dopamine agonists, muscle relaxants, opioids and overactive bladder medications). Outcomes are expressed with patients receiving a lower dose of anti-depressant as the referent group. We then tested for statistical interactions to determine whether the association between anti-depressant drug dose (higher *vs.* lower) and outcome was modified by the presence of moderate CKD. All odds ratios were interpreted as relative risks (appropriate given the low incidences observed). We conducted all statistical analyses using SAS, version 9.3.

## Results

The study included 117,811 eligible older adults in routine outpatient care who were dispensed a new oral study anti-depressant (patient selection diagram presented in Additional file 1: Figure S1). A total of 36,651 patients (31%) were initiated on a higher anti-depressant dose, and 81,160 patients (69%) were initiated on a lower anti-depressant dose. Most prescriptions were written by a primary care physician (76.8%). There was no significant difference in 20 of the 25 measured baseline characteristics between the higher and lower anti-depressant dose groups (Table 2). However, compared to those in the lower dose group, patients in the higher dose group were more likely to be prescribed mirtazapine and venlafaxine (26.0% *vs.* 18.6%, and 62.5% *vs.* 29.8%, respectively), were more likely to be younger (mean 73.7 years *vs.* 75.6 years), were more likely to enter the cohort between the years 2006 to 2009 (40.0% *vs.* 34.5%), were less likely to enter the cohort between the years 2002 to 2005 (44.8% *vs.* 52.7%) and were less likely to be women (61.3% *vs.* 67.3%).

**Table 2:** Baseline characteristics by anti-depressant dose

	**Higher dose**	**Lower dose**	**Standardized difference**
	**N = 36,651**	**N = 81,160**	
**Anti-depressant type, *n (%)***			
Paroxetine	4,216 (11.5)	41,889 (51.6)	0.96
Mirtazapine	9,531 (26.0)	15,101 (18.6)	0.18
Venlafaxine	22,904 (62.5)	24,170 (29.8)	0.69
**Demographics**			
Age, years, *mean (SD)*	73.7 (6.8)	75.6 (5.8)	0.29
Women, *n (%)*	22,472 (61.3)	54,631 (67.3)	0.13
**Year of cohort entry, *n (%)***			
2002-2005	16,434 (44.8)	4,2770 (52.7)	0.16
2006-2009	14,661 (40.0)	2,7984 (34.5)	0.11
2010-2011	5,556 (15.2)	10,406 (12.8)	0.07
**Income quintile, *n (%)***			
First (lowest)	7,663 (20.9)	16,725 (20.6)	0.01
Second	7,552 (20.6)	17,627 (21.7)	0.03
Third (middle)	7,034 (19.20	16,003 (19.7)	0.01
Fourth	7,118 (19.4)	15,226 (18.8)	0.02
Fifth (highest)	7,142 (19.5)	15,327 (18.9)	0.02
Missing	142 (0.4)	252 (0.3)	0.01
**Rural Location, *n (%)***	5,386 (14.7)	10,943 (13.5)	0.03
**Modified Charlson score**^**Ŧ**^**, *n (%)***			
0 or no hospitalization	25,360 (69.2)	56,063 (69.0)	0.00
1	4,263 (11.6)	9,548 (11.7)	0.00
2	3,514 (9.6)	7,571 (9.3)	0.01
≥3	3,514 (9.6)	8,005 (9.9)	0.01
**Co-morbidities**^**£**^**, *n (%)***			
Chronic liver disease	1,348 (3.7)	2,970 (3.7)	0.00
Chronic kidney disease*	1,609 (4.4)	3,772 (4.7)	0.01
Chronic obstructive pulmonary disease	1,849 (5.0)	4,389 (5.4)	0.02
Coronary artery disease^¶^	12,951 (35.3)	31,350 (38.6)	0.07
Heart failure	4,133 (11.3)	10,715 (13.2)	0.06
Stroke/Transient ischemic attack	1,148 (3.1)	2,625 (3.2)	0.01
Diabetes mellitus (on medication)^+^	5,878 (16.0)	12,203 (15.0)	0.03
**Medication**^**¥**^**, *n (%)***			
Anticonvulsants	3,728 (10.2)	6,574 (8.1)	0.07
Gabapentin	363 (1.0)	525 (0.7)	0.04
Antipsychotics	2,499 (6.8)	3,752 (4.6)	0.09
Barbiturates	89 (0.2)	163 (0.2)	0.01
Benzodiazepines	15,736 (42.9)	35,681 (44.0)	0.02
Histamine2-receptor antagonists	3,417 (9.3)	9,764 (12.0)	0.09
Dopamine agonists	298 (0.8)	517 (0.6)	0.02
Muscle relaxants	311 (0.9)	564 (0.7)	0.02
Opioids	10,408 (28.4)	21,632 (26.7)	0.04
Overactive bladder medications	1,424 (3.9)	2,992 (3.7)	0.01
**Prescribing physician, *n (%)***			
General practitioner	27,884 (76.1)	62,622 (77.2)	0.03
Internist	251 (0.7)	669 (0.8)	0.02
Neurologist	179 (0.5)	408 (0.5)	0.00
Other	3,514 (9.6)	6,354 (7.8)	0.06
Missing	4,823 (13.2)	11,107 (13.7)	0.02

^£^Co-morbidities were assessed in the five years prior to the index date using International Classification of Diseases 9^th^ revision (ICD-9) and 10^th^ revision (ICD-10) codes.

^Ŧ^Assessed with an algorithm using diagnosis codes from hospitalizations in the five years prior; patients with no hospitalizations during this period were given a value of zero.

*Identified individuals with CKD using an algorithm of diagnosis codes validated in our region for older adults [32].

The algorithm identified patients with a median estimated glomerular filtration rate (eGFR) of 38 mL/min per 1.73 m^2^ (interquartile range 27 to 52), whereas its absence identified patients with a median eGFR of 69 mL/min per 1.73 m^2^ (interquartile range 56 to 82).

^¶^Coronary artery disease includes receipt of coronary artery bypass graft surgery, percutaneous coronary intervention and diagnoses of angina.

^¥^Medication use was assessed in the 180 days prior to the index date.

+Diabetic medications include oral hypoglycemics and insulin.

### 30-day hospitalization with urgent head Computed Tomography (CT) scan

**Figure 1:**
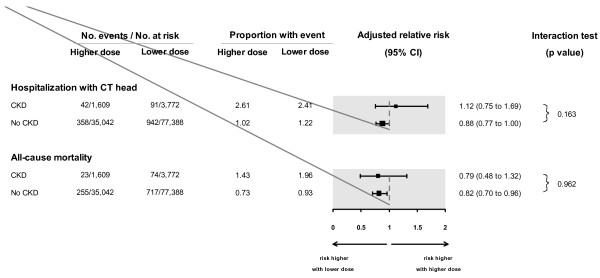
**Association between anti-depressant dose and adverse events in patients with and without Chronic Kidney Disease (CKD; where CKD was defined by diagnosis codes).** There was no difference in association between anti-depressant dose and adverse events in patients with and without CKD (interaction p-value 0.163 and 0.962, respectively for each outcome). Estimates of risks for patients without CKD (n = 112,430) were more precise than for patients with CKD (n = 5,381). Adjusted for 15 covariates: antidepressant type, age, sex, year of cohort entry, modified Charlson score (a co-morbidity index), and concurrent medication use (anticonvulsants, gabapentin, antipsychotics, barbiturates, benzodiazepines, histamine2-receptor antagonists, dopamine agonists, muscle relaxants, opioids and overactive bladder medications). Abbreviations: CI, confidence interval; CT, computed tomography.

**Figure 2:**
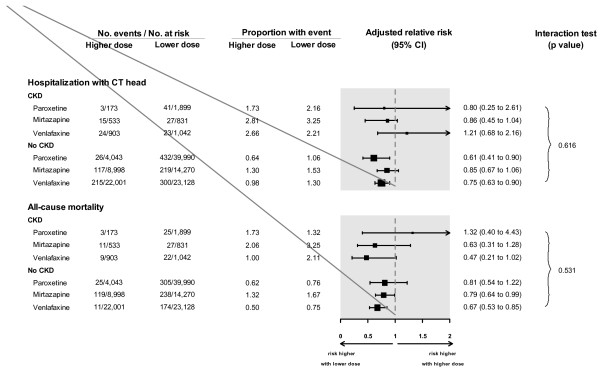
**Association between anti-depressant dose and adverse events in patients with and without Chronic Kidney Disease (CKD) stratified by the type of anti-depressant.** With a smaller number of patients and events, estimates of risks for patients with CKD were less precise than for patients without CKD. Stratified models by antidepressant type adjusted for 14 covariates: age, sex, year of cohort entry, modified Charlson score (a co-morbidity index), and concurrent medication use (anticonvulsants, gabapentin, antipsychotics, barbiturates, benzodiazepines, histamine2-receptor antagonists, dopamine agonists, muscle relaxants, opioids and overactive bladder medications). Abbreviations: CI, confidence interval; CT, computed tomography.

Initiation of a higher *vs.* lower anti-depressant dose was associated with a lower risk of hospitalization with head CT scan (Table 3; 400/36,651 [1.09%] *vs.* 1,033/81,160 [1.27%], unadjusted relative risk 0.86 [95% CI 0.76 to 0.96]). After adjusting for 15 potential confounders the association was no longer significant (Table 3; adjusted relative risk 0.90 [95% CI 0.80 to 1.02]). The association was not appreciably different in patients with and without moderate CKD (when the presence of this condition was assessed with diagnosis codes; Figure 1; interaction *P* value = 0.16). The association was also not appreciably different in patients with and without moderate CKD when examined separately by each of the three anti-depressant drugs (recognizing stratifying the results in this way resulted in smaller sample sizes and wider confidence intervals; Figure 2).

**Table 3:** Association between anti-depressant dose and 30-day outcomes

	**Number of events, *n (%)***		**Relative risk (Unadjusted)**	**Relative risk (Adjusted)**	**Absolute risk reduction (%)**
	**Higher dose**^**£**^	**Lower dose**^¶^	**(95% CI)**	**(95% CI)**^**¥**^	
					**(95% CI)**
	**N = 36,651**	**N = 81,160**			
**Hospital admission with head CT scan**	400 (1.09)	1,033 (1.27)	0.86 (0.76 – 0.96)	0.90 (0.80 – 1.02)	(…)
**All-cause mortality**	278 (0.76)	791 (0.97)	0.78 (0.68 – 0.89)	0.82 (0.71 – 0.95)	0.22 (0.10 – 0.33)

^£^Higher dose of anti-depressant defined as > 20 mg/day of paroxetine, > 20 mg/day of mirtazapine, or > 37.5 mg/day of venlafaxine.

^¶^Lower dose of anti-depressant defined as ≤ 20 mg/ day of paroxetine, ≤ 20 mg/day of mirtazapine, or ≤ 37.5 mg/day of venlafaxine.

^¥^Adjusted for 15 covariates (see Methods).

(…) Absolute risk difference was not calculated as adjusted relative risk was not statistically significant for this outcome.

Patients prescribed the lower anti-depressant dose served as the referent group.

*Abbreviations: CI* confidence interval, *CT* computed tomography.

### 30-day all-cause mortality

Initiation of a higher *vs.* lower anti-depressant dose was associated with a lower risk of 30-day all-cause mortality (Table 3; 278/36,651 [0.76%] *vs.* 791/81,160 [0.97%], absolute risk reduction 0.22% [95% CI 0.10% to 0.33%], unadjusted relative risk 0.78 [95% CI 0.68 to 0.89]). Adjusting for 15 potential confounders had no appreciable impact on this observed association (adjusted relative risk 0.82 [95% CI 0.71 to 0.95; Table 3). The association was not appreciably different in patients with and without moderate CKD (when the presence of this condition was assessed with diagnosis codes; Figure 1; interaction *P* value = 0.96). The association was also not appreciably different in patients with and without moderate CKD when examined separately by each of the three anti-depressant drugs (Figure 2).

### Additional analyses

**Figure 3:**
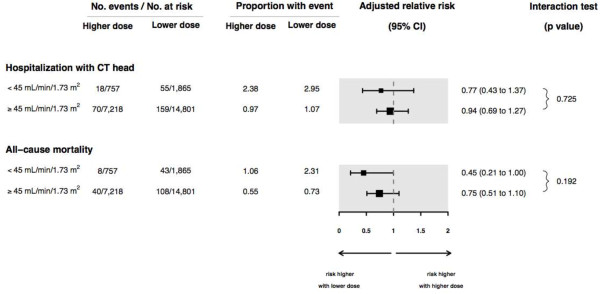
**Association between anti-depressant dose and adverse events in patients with and without Chronic Kidney Disease (CKD) where the presence of CKD was defined by an estimated glomerular filtration rate (eGFR) < 45 mL/min per 1.73 m**^**2**^**.** All point estimates of the relative risk with a higher *vs.* lower anti-depressant dose and outcomes were below a value of 1 in patients with and without CKD, with the association not appreciably different in those with and without CKD. Adjusted for 15 covariates: antidepressant type, age, sex, year of cohort entry, modified Charlson score (a co-morbidity index), and concurrent medication use (anticonvulsants, gabapentin, antipsychotics, barbiturates, benzodiazepines, histamine2-receptor antagonists, dopamine agonists, muscle relaxants, opioids and overactive bladder medications). Abbreviations: CI, confidence interval; CT, computed tomography.

We repeated our analysis in a subpopulation of older adults with baseline serum creatinine values (n = 24,641) comparing a higher *vs.* lower anti-depressant dose and each of our two outcomes. In this analysis the clinically important threshold used to define moderate CKD was an eGFR < 45 mL/min per 1.73 m^2^ (as higher eGFR thresholds (45 to 60 mL/min per 1.73 m^2^) may not identify substantial CKD in the elderly, and choosing a lower eGFR threshold (<30 mL/min per 1.73 m^2^) meant less patients (n = 587 for this analysis)). Results are presented in Figure 3. Point estimates of the relative risk of a higher *vs.* lower anti-depressant dose and outcomes of head CT and all-cause mortality were all below a value of 1 in patients with and without a baseline eGFR value < 45 mL/min per 1.73 m^2^. The association was not appreciably different in those with and without moderate CKD (Figure 3; interaction *P* value = 0.73 and 0.19 for each outcome, respectively).

In the primary cohort we considered the outcome of hospitalization using diagnostic codes for delirium (recognizing the coding for this outcome is insensitive and underestimates events but was expected not to operate differently in the two anti-depressant dose groups). Initiation of a higher *vs.* lower anti-depressant dose was not associated with a difference in risk of hospitalization with delirium (43/36,651 [0.16%] *vs.* 126/81,160 [0.12%], unadjusted relative risk 0.76 [95% CI 0.53 to 1.07]). The association was not different in patients with and without moderate CKD (interaction *P* value = 0.75).

## Discussion

We report the 30-day risk of two major adverse events as assessed in large healthcare administrative databases in older adults who initiated a higher *vs.* lower dose of one of three common anti-depressants used in routine outpatient care. Contrary to our expectation, compared to a lower dose, initiation of a higher dose of anti-depressant was associated with a lower 30-day risk of all-cause mortality. This association appeared similar in patients with and without moderate CKD, although estimates in patients with CKD were less precise. There was no association between the anti-depressant dose and the risk of hospitalization with urgent neuroimaging, an extreme outcome in the spectrum of delirium.

It is well established that older individuals are more prone to adverse drug reactions [8,9,33]. We hypothesized that the presence of moderate CKD would make older adults particularly vulnerable to toxicity from higher doses of three study anti-depressants that have been shown to accumulate in CKD (paroxetine, mirtazapine, venlafaxine). The data supporting dose reductions of these anti-depressants comes from pharmacokinetic studies and case studies [6,12]. To our knowledge, our study is the first to examine the clinical consequences of failing to dose adjust anti-depressants in the setting of moderate CKD at the population level. There is a need to determine if drug dosing decisions based on pharmacokinetic data influence real practice outcomes. The lower risk of death we observed with higher doses of anti-depressant was contrary to our hypothesis. It is possible the association relates to unmeasured confounding between the two dosing groups. It is also possible that higher *vs.* lower doses of anti-depressants are more efficacious in treating depression and improve survival. Inadequately treated depression can increase mortality risk through nonadherence to medications and health care appointments, poor nutrition, lack of social support, increased inflammation and compromised immunity [37-40]. Examining the causes of death such as suicide or withdrawal of care in this context would be useful in future studies, as such information was not reliable in our data sources.

With respect to patient safety, if concerns remain about the use of certain anti-depressants in patients with moderate CKD, a reasonable alternative is to prescribe an anti-depressant such as fluoxetine where the pharmacokinetics are not altered by the presence of CKD. Observational data also suggests that this anti-depressant is well tolerated by patients with CKD [14].

Our study has several strengths. The use of Ontario’s broadly inclusive healthcare databases provided us with a large representative sample. The data was complete, drug dose and outcomes were accurately recorded, and patient loss to follow-up was minimal (emigration in our region is less than 1% per year) [41].

Our study has several limitations. The similarity of measured patient baseline characteristics in our two anti-depressant dose groups helped reduced concerns about the influence of confounding. The concern over residual confounding was also reduced by the lack of substantial change in the observed association after adjustment for multiple potential confounders. However, as mentioned, as in any observational study the possibility of unmeasured confounding can never be completely eliminated. We knew the anti-depressant was dispensed by a pharmacy but had no information on compliance. Future studies should include more patients with very low levels of eGFR (i.e. < 30 mL/min per 1.73 m^2^), and should also collect body weight so that eGFR can also be expressed in mL/min. Our primary outcome was assessed retrospectively using existing healthcare database records and relied on urgent neuroimaging as a proxy for the diagnosis of delirium, which is an insensitive method to identify important changes in cognition. Rather, if resources allow for it, a well-designed prospective study with independent outcome adjudication would more precisely capture benefits and risks associated with initiating a higher *vs.* lower anti-depressant dose.

## Conclusion

In this study which used large healthcare administrative databases, contrary to our hypothesis we failed to observe an association between initiation of a study anti-depressant at a higher dose and a higher risk of two adverse outcomes in older adults with moderate CKD.

## Competing interests

The authors declare that they have no relevant financial interests. The Institute for Clinical Evaluative Sciences (ICES) is a non-profit research corporation funded by an annual grant from the Ontario Ministry of Health and Long-Term Care (MOHLTC). The research was conducted at the ICES Western facility, which receives financial support from the Academic Medical Organization of Southwestern Ontario, the Schulich School of Medicine and Dentistry at Western University and the Lawson Health Research Institute. The opinions, results and conclusions are those of the authors and are independent from the funding sources. No endorsement by ICES or the Ontario MOHLTC is intended or should be inferred.

## Authors’ contributions

VD drafted the manuscript and performed literature reviews. SD carried out and supervised the statistical analyses. JF also aided in preparing the manuscript and data analysis. SJ aided in planning and carrying out analyses. TG, ZH, AJ and MW aided in manuscript preparation, study coordination, and statistical modeling. SS and DT aided in study design, research methodology and interpretation of data. AG conceived of the study, and participated in its design, coordination and manuscript preparation. All authors read and approved the final manuscript.

## Pre-publication history

The pre-publication history for this paper can be accessed here:

http://www.biomedcentral.com/1471-2369/15/79/prepub

## Supplementary Material

Additional file 1: Figure S1 Patient Selection. **Table S1.** Checklist of Recommendations for Reporting of Observational Studies Using the STROBE Guidelines. **Table S2.** Coding Definitions for Demographics, Comorbid Conditions and Outcomes. Click here for file
