# Supplementary material for: Higher anti-depressant dose and major adverse outcomes in moderate chronic kidney disease: a retrospective population-based study
Source: BMC Nephrol. 2014 May 10;15:79. doi: 10.1186/1471-2369-15-79 (PMC4024017; doi:10.1186/1471-2369-15-79)
Supplement: Additional file 1: Figure S1 — Patient Selection. Table S1. Checklist of Recommendations for Reporting of Observational Studies Using the STROBE Guidelines. Table S2. Coding Definitions for Demographics, Comorbid Conditions and Outcomes. [file 1471-2369-15-79-S1.doc]

Additional file 1

Figure S1 **Patient Selection**

Patients with evidence of a new outpatient oral prescription for a study anti-depressant between April 1^st^ 2002 and December 31^st^ 2011.

N = 169,435

Patients excluded from study: (N = 51,624)

Age <66 on the prescription date: N =12,588

Hospital discharge in 2 days prior to their prescription date: N = 3,833

Long-term care home residents: N = 30,360

End-stage renal disease before prescription date: N = 1,522

More than one type of anti-depressant on prescription date: N = 3,321

Patients included in final cohort

N = 117,811

N = 36,651 Higher dose

N = 81,160 Lower dose

Subpopulation with baseline serum creatinine levels

N = 24,641

N = 7,975 Higher dose

N = 16,666 Lower dose

Table S1 **Checklist of Recommendations for Reporting of Observational Studies Using the STROBE Guidelines**

|  | Item No | Recommendation | Reported |
| --- | --- | --- | --- |
| **Title and abstract** | 1 | (a) Indicate the study’s design with a commonly used term in the title or the abstract | Abstract |
|  |  | (b) Provide in the abstract an informative and balanced summary of what was done and what was found | Abstract |
| Introduction | | |  |
| Background/rationale | 2 | Explain the scientific background and rationale for the investigation being reported | Introduction |
| Objectives | 3 | State specific objectives, including any prespecified hypotheses | Introduction |
| Methods | | |  |
| Study design | 4 | Present key elements of study design early in the paper | Methods – setting and study design |
| Setting | 5 | Describe the setting, locations, and relevant dates, including periods of recruitment, exposure, follow-up, and data collection | Methods – setting and study design; data sources |
| Participants | 6 | (a) Give the eligibility criteria, and the sources and methods of selection of participants. Describe methods of follow-up | Methods - participants |
|  |  | (b)For matched studies, give matching criteria and number of exposed and unexposed | not applicable |
| Variables | 7 | Clearly define all outcomes, exposures, predictors, potential confounders, and effect modifiers. Give diagnostic criteria, if applicable | Methods – anti-depressant dosing; outcomes |
| Data sources/ measurement | 8 | For each variable of interest, give sources of data and details of methods of assessment (measurement). Describe comparability of assessment methods if there is more than one group | Methods – data sources |
| Bias | 9 | Describe any efforts to address potential sources of bias | Methods – statistical analysis; Discussion |
| Study size | 10 | Explain how the study size was arrived at | not applicable; use of existing health records |
| Quantitative variables | 11 | Explain how quantitative variables were handled in the analyses. If applicable, describe which groupings were chosen and why | not applicable |
| Statistical methods | 12 | (a) Describe all statistical methods, including those used to control for confounding | Methods – statistical analysis |
|  |  | (b) Describe any methods used to examine subgroups and interactions | Methods – participants; statistical analysis |
|  |  | (c) Explain how missing data were addressed | not applicable |
|  |  | (d) If applicable, explain how loss to follow-up was addressed | not applicable |
|  |  | (e) Describe any sensitivity analyses | Results – additional analyses |
| Results | | |  |
| Participants | 13 | (a) Report numbers of individuals at each stage of study—e.g. numbers potentially eligible, examined for eligibility, confirmed eligible, included in the study, completing follow-up, and analysed | Results; Appendix A |
|  |  | (b) Give reasons for non-participation at each stage | Appendix A |
|  |  | (c) Consider use of a flow diagram | Appendix A |
| Descriptive data | 14 | (a) Give characteristics of study participants (e.g. demographic, clinical, social) and information on exposures and potential confounders | Methods – participants; Results, Table 2 |
|  |  | (b) Indicate number of participants with missing data for each variable of interest | Complete with exception of specialty of prescribing physician and income quintile (missing data described in Table 2) |
|  |  | (c) Summarise follow-up time (e.g. average and total amount) | not applicable |
| Outcome data | 15 | Report numbers of outcome events or summary measures over time | Results; Table 3; Appendix C |
| Main results | 16 | (a) Give unadjusted estimates and, if applicable, confounder-adjusted estimates and their precision (e.g. 95% confidence interval). Make clear which confounders were adjusted for and why they were included | Results; Table 3; Appendix C |
|  |  | (b) Report category boundaries when continuous variables were categorized | not applicable |
|  |  | (c) If relevant, consider translating estimates of relative risk into absolute risk for a meaningful time period | Results; Table 3; Appendix C |
| Other analyses | 17 | Report other analyses done—e.g. analyses of subgroups and interactions, and sensitivity analyses | Results; Figure 1; Appendix C |
| Discussion | | |  |
| Key results | 18 | Summarise key results with reference to study objectives | Discussion |
| Limitations | 19 | Discuss limitations of the study, taking into account sources of potential bias or imprecision. Discuss both direction and magnitude of any potential bias | Discussion |
| Interpretation | 20 | Give a cautious overall interpretation of results considering objectives, limitations, multiplicity of analyses, results from similar studies, and other relevant evidence | Discussion |
| Generalisability | 21 | Discuss the generalisability (external validity) of the study results | Discussion |
| Other information | | |  |
| Funding | 22 | Give the source of funding and the role of the funders for the present study and, if applicable, for the original study on which the present article is based | Cover page |

| ***Table S2* Coding Definitions for Demographics, Comorbid Conditions and Outcomes** | | |
| --- | --- | --- |
| **Characteristic / Outcome** | **Database** | **Codes** |
| Age | RPDB |  |
| Sex | RPDB |  |
| Socioeconomic Status | Statistics Canada |  |
| Long Term Care Facility Utilization | ODB |  |
| Rural Location | Statistics Canada |  |
| Chronic Kidney Disease | CIHI DAD  OHIP | ICD-9: 4030, 4031, 4039, 4040, 4041, 4049, 585, 586, 5888, 5889  ICD-10: E102, E112, E132, E142, I12, I13, N08, N18, N19  403, 585 |
| Renal Transplant | CIHI DAD  OHIP | ICD-9: V420, 99681  ICD-10: T861, N165, Z940  CCP: 6743, 675  CCI: 1PC85  E762, S435, E769, S434, E771, Z631, G347, G348, G412, G408, G409 |
| Dialysis | CIHI DAD  OHIP | ICD-9: V451, V560, V568, 36104  ICD-10: T824, Y602, Y612, Y622, Y841, Z49, Z992, N180, E1022, E1023,E1122, E1123, E1322, E1323, E1422, E1423  CCP: 5127, 5142, 5143, 5195, 6698  CCI: 1OT53DATS, 1OT53HATS, 1OT53LATS, 1SY55LAFT, 7SC59QD, 1KY76, 1PZ21  R850, G324, G336, G327, G862, G865, G099, R825, R826, R827, R833, R840, R841, R843, R848, R851, Z450, Z451, Z452, G864, R852, R853, R854, R885, G333, H540, H740, R849, G323, G325, G326, G860, G863, G866, G330, G331, G332, G861, G082, G083, G085, G090, G091, G092, G093, G094, G095, G096, G294, G295 |
| Computed Tomography Head | CIHI DAD  OHIP | CCI: 3AN20, 3EA20, 3ER20  X188, X400, X401, X402, X405, X408 |
| Stroke | CIHI DAD | ICD-9: 434, 436, 431, 4358, 4359  ICD-10: H341, I630 I631, I632, I633, I634, I635, I638, I639, I629, I64, G45, I61 |
| Congestive Heart Failure | CIHI DAD  OHIP | ICD-9: 425, 5184, 514, 428  ICD-10: I500, I501, I509, I255, J81  CCP: 4961, 4962, 4963, 4964  CCI: 1HP53, 1HP55, 1HZ53GRFR, 1HZ53LAFR, 1HZ53SYFR  R701, R702, Z429, 428 |
| Coronary Artery Disease | CIHI DAD  OHIP | ICD-9: 412, 410, 413, 414, 4292, 4295, 4296, 4297  ICD-10: I20, I21, I22, I23, I24, I25, Z955, Z958, Z959, R931, T822  CCI: 1IJ26, 1IJ27, 1IJ54, 1IJ57, 1IJ50, 1IJ76  CCP: 4801, 4802, 4803, 4804, 4805, 481, 482, 483  R741, R742, R743, G298, E646, E651, E652, E654, 410, 412, 413 |
| Chronic Obstructive Pulmonary Disease | CIHI DAD | ICD-9: 491, 492, 496  ICD-10: J41, J43, J44 |
| Chronic Liver Disease | CIHI DAD  OHIP | ICD-9: 4561, 4562, 070, 5722, 5723, 5724, 5728, 573, 7824, V026, 2750, 2751, 7891, 7895, 571  ICD-10: B16, B17, B18, B19, I85, R17, R18, R160, R162, B942, Z225, E831, E830, K70, K713, K714, K715, K717, K721, K729, K73, K74, K753, K754, K758, K759, K76, K77  571, 573, 070, Z551, Z554 |
| Medication Use | ODB |  |
| Depression | CIHI DAD | ICD-10: F32, F33 |
| Delirium | CIHI DAD | ICD-10: F050, F058, F059 |
| All Cause Mortality | RPDB |  |
| Proteinuria | Gamma Dynacare  CERNER |  |
| GFR | Gamma Dynacare  CERNER |  |
| Serum Creatinine | Gamma Dynacare  CERNER |  |

CCI = Canadian Classification of Health Interventions; CCP = Canadian Classification of Diagnostic, Therapeutic and Surgical Procedures; CIHI – DAD = Canadian Institute for Health Information Discharge Abstract Database; ICD-9 = International Classification of Disease, Ninth Revision; ICD-10 = International Classification of Diseases, Tenth Revision; ODB = Ontario Drug Benefits; OHIP = Ontario Health Insurance Plan; RPDB = Registered Persons Database of Ontario
